# Advances in pediatrics in 2017: current practices and challenges in allergy, endocrinology, gastroenterology, genetics, immunology, infectious diseases, neonatology, nephrology, neurology, pulmonology from the perspective of Italian Journal of Pediatrics

**DOI:** 10.1186/s13052-018-0524-7

**Published:** 2018-07-17

**Authors:** Carlo Caffarelli, Francesca Santamaria, Dora Di Mauro, Carla Mastrorilli, Silvia Montella, Bertrand Tchana, Giuliana Valerio, Alberto Verrotti, Mariella Valenzise, Sergio Bernasconi, Giovanni Corsello

**Affiliations:** 1 Clinica Pediatrica, Department of Medicine and Surgery, Azienda Ospedaliera-Universitaria, University of Parma, Parma, Italy; 2 Department of Translational Medical Sciences, Federico II University, Naples, Italy; 3 Cardiologia Pediatrica, Azienda Ospedaliera-Universitaria, Parma, Italy; 4 Pediatria, Dipartimento di Scienze Motorie e del Benessere, Università di Napoli Parthenope, Naples, Italy; 5 Department of Pediatrics, University of L’Aquila, L’Aquila, Italy; 6 UOC Clinica Pediatrica AOU G, Martino Università di Messina, Messina, Italy; 7 Pediatrics Honorary Member University Faculty, G D’Annunzio University of Chieti-Pescara, Chieti, Italy; 8 Department of Sciences for Health Promotion and Mother and Child Care “G. D’Alessandro”, University of Palermo, Palermo, Italy

**Keywords:** Allergy, Endocrinology, Gastroenterology, Genetics, Immunology, Infectious diseases, Neonatology, Nephrology, Neurology Pediatrics, Pulmonology

## Abstract

This review provides an overview of a remarkable number of significant studies in pediatrics that have been published over the past year in the Italian Journal of Pediatrics. We have selected information from papers presented in the Journal that deal with allergy, endocrinology, gastroenterology, genetics, immunology, infectious diseases, neonatology, nephrology, neurology, pulmonology. The relevant epidemiologic findings, and developments in prevention, diagnosis and treatment of the last year have been discussed and placed in context. We think that advances achieved in 2017 will help readers to make the future of patients better.

## Background

A remarkable number of papers that may have a significant impact on the management of different childhood diseases, have been issued in Italian Journal of Pediatrics in 2017. This review highlights the most accessed articles and it puts them in the specific context. We have covered a choice of articles on allergy, endocrinology, gastroenterology, genetics, immunology, infectious diseases, neonatology, nephrology, neurology, pulmonology in childhood.

## Review

### Allergy and immunology

#### 1-diagnosis of cow’s milk allergy; 2 -corticosteroids in atopic dermatitis; 3-innovative treatments for allergic diseases; 4-ANCA-associated vasculitis

Clinical features of immediate IgE-mediated reactions [1] triggered by cow’s milk include skin symptoms (rash, angioedema, urticaria) [2], nausea, vomiting, abdominal pain, diarrhea, rhinoconjunctivitis, asthma, anaphylaxis. The diagnosis of cow’s milk allergy is based on a convincing history and positive IgE tests (skin prick tests (SPTs) and/or serum specific IgE antibodies [3]), but the gold standard is a positive oral food challenge [4]). A systematic review [5] has addressed the utility of SPT or IgE cut-off values for avoiding cow’s milk challenge. In children < 2 years of age, an IgE cut-off point of 5 kU/L and SPT cut-offs for weal diameter > 6 mm for commercial extract and > 8 mm for fresh milk were found. No cut-off values were found to predict allergy to fresh cow’s milk and baked milk in children > 2 years of age. Cut-off values of IgE tests are affected not only by age but also by several factors such as studied population, clinical picture, time of investigation, methods of IgE testing. So, it is advisable that reliable cut-off values should be set up by each allergist using data from his laboratory. Patch test to cow’s milk is not useful [6].

Many efforts have been dedicated to improve the treatment of atopic diseases. The pharmacological first-line treatment for atopic dermatitis is based on topical corticosteroids (TCS) [7], even if a systemic inflammatory response is often present [8]. Poor adherence and lack of effectiveness of treatment is commonly caused by steroid fear [9]. By using a questionnaire, El Hachem M. et al. [10] investigated “corticophobia” in families of 300 patients. Eighty-one percent of the respondents reported to be afraid of TCS. Most care givers were concerned that danger of TCS was not related to side effect and that receives help from treatment do not overcome detriments. There was also fear to incorrectly apply an excessive quantity of cream. The result of this study highlights the need for age related educational programs for the control of atopic dermatitis. Such programs have been shown to be effective in ameliorating the disease [11] and in improving the quality of life of eczematous children [12].

Allergen-specific immunotherapy (AIT) is considered the only causal disease-modifying treatment for allergic disorders. Among the 2017 highlights is a consensus [13] that provides recommendations for use of AIT in practice. This consensus emphasized that selection of patients and schedule of administration are a crucial issue for efficacy of AIT. In children with allergic rhinitis and allergic asthma, both subcutaneous and sublingual AIT have very good efficacy and safety. Furthermore, in children with rhinoconjunctivitis due to grass allergy, AIT is useful for reducing the risk for asthma development. However, it is unclear whether it may reduce the onset of pollen-food syndrome that is a frequent result of cross-reactivity between pollen allergens and food allergens with similar structure [14]. It should be underlined that choice of airborne allergens for AIT should be based on diagnosis of sensitization to relevant genuine molecules rather than to panallergens [15]. Subcutaneous AIT is recommended for allergy to hymenoptera venom. Oral AIT has shown to be a promising treatment for IgE-mediated food allergy while there are contrasting data on the role of AIT in atopic dermatitis [16].

Another area of research has been the treatment of allergic rhinitis with probiotics. Probiotics may change immune function by upregulating Th1 pathway and refurbishing T regs [17]. Moreover, probiotics downregulate Th2 cytokines in allergic rhinitis. [18]. Probiotics may also prevent mucosal damage induced by hydrogen peroxide [19] which is increased during exacerbations of respiratory allergy [20]. Del Giudice et al. [21] showed that a Bifidobacteria mixture, *B longum*, *B infantis* and *B breve*, significantly reduced nasal symptom score and improve quality of life in children with parietaria-induced allergic rhinitis and was well tolerated. This investigation supports earlier observations of a possible benefit of adjuvant therapy with probiotics in seasonal allergic rhinitis [22].

Data on anti-neutrophil cytoplasmic antibody (ANCA)-associated vasculitis (AVA) are scarce in childhood perhaps due to rarity in pediatric age, 0.5–6 new cases /million children/year, in comparison with adulthood, but possibly also due to lack of clinical studies and investigations on natural history. With this in mind, Calatroni’s overview of childhood-onset AVA is relevant [23]. Fever, fatigue, anorexia, and weight loss precede systemic manifestations. So, early diagnosis is difficult. In microscopic polyangiitis (MPA), prognosis mainly depends on renal symptoms that are present in almost all cases at onset of disease, and on lung manifestations. Granulomatosis with polyangiitis (GPA, formerly Wegener’s granulomatosis) [24] is characterized by ear, nose and throat manifestations. Involvement of kidney and lower respiratory tract is less frequent. Clinical phenotypes in childhood and adulthood are similar. However, at variance from adults, children frequently present subglottic stenosis and saddle nose deformity, while peripheral neuropathy and cardiovascular involvement are rare. Eosinophilic granulomatosis with polyangiitis (EGPA, formerly Churg-Strauss syndrome) usually starts with asthma, allergic rhinitis, and sinusitis. Later features are hypereosinophilia with nasal polyps, and lung infiltrates, and vasculitis with urticarial rash and purpura, and cardiovascular, musculoskeletal, gastrointestinal, neurologic, and renal manifestations. ANCA positivity is reported in 50–60% of children with MPA and GPA but only in 25% of children with EGPA. Children are treated with glucocorticosteroids plus cyclophosphamide as with adults [25]. Rituximab is for non-responsive or relapsing children. Other options are mycophenolate, methotrexate, and plasma exchange. Maintenance treatment includes azathioprine, low-dose rituximab or mycophenolate. Mortality rate is 5–10% in children and it is lower than in adults [26].

### Endocrinology

#### 1-obesity; 2-Suclinical hypothyroidism

Pediatric obesity is associated with multiple physical and psychosocial co-morbidities [27–29] and with premature morbidity and mortality for metabolic disease and cardiovascular events, which are the leading cause of mortality in the industrialized countries [30–32]. A few studies analyzed the presence of cardiometabolic risk factors (CMRFs) in the very first years of life [33, 34]. CMRFs, including fatty liver, were already detectable in preschoolers with overweight or obesity, despite their short-term exposure to excess weight [34]. Riaño-Galán et al. [35] showed higher lipid risk profiles in four-year-old overweight/obese children, in whom weight status was also associated to maternal prepregnancy body mass index (BMI), gestational diabetes, and birth weight. These findings support the need of prevention actions based on improving diet quality and increasing physical activity in women both preconceptionally and during pregnancy. Additionally, it is recommended identifying children at risk for obesity as early as possible in the postnatal life and avoiding excessive weight gain and/or increased weight-to-length ratio since the very first months of life [36]. For preventive purposes, it is necessary to rely upon a classification system with a greater sensitivity in identifying children and adolescents with overweight and obesity. Valerio et al. [37] compared the ability of different BMI reference systems in estimating overweight or obesity children with CMRFs among the BMI references by the Italian Society for Pediatric Endocrinology and Diabetology and those from the World Health Organization (WHO), and the International Obesity Task Force. WHO thresholds had the highest sensitivity in identifying obese subjects with clustered CMRFs. This study arises the question whether the WHO standards should replace the national standards for clinical practice and obesity screening. That is not of secondary importance if we consider that even a BMI within the accepted normal range, i.e. in the 50th–74th percentiles during adolescence, was associated with increased cardiovascular and all-cause mortality in adulthood [31].

Furthermore, great attention has recently been turned to severe obesity in children, which affects 4–6% of all youth in the United States and 2.7% children in Italy [38, 39]. Several studies indeed showed that the prevalence of CMRFs, psychosocial functioning, bullying experiences and quality of life rises with greater degrees of obesity [37–40].

All these evidences reinforce the issue that pediatric obesity is a real chronic condition that needs to be effectively prevented. When obesity is established, there is a need for early identification and treatment of children who are at the highest risk of developing the obesity-related complications. Further studies are necessary to assess whether the screening for cardiometabolic abnormalities should be anticipated at an earlier age than is now recommended.

In addition to the obesity-related potentially life-threatening disease [41–43], over the last decades increased attention has been focused on the possible causal relationship between obesity and migraine. Studies in adults have shown that obese patients are more likely to have migraine that non-obese individuals and that obesity is associated to higher frequency and severity of headache attacks among patients suffering from migraine [44–46]. Comparable results have been reached in children [47–54]. Based on these observations, Verrotti et al. [55] investigated the impact of a weight loss program on migraine outcome. Interestingly they found significant improvements in both adiposity and headache frequency and intensity. Later, the same cohort of patients was enrolled for a second study aimed at identifying possible metabolic parameters associated with good migraine control. Authors reported that not migraine-free patients showed higher adiposity values and that insulin resistance status was associated with a 3.5 time the odds of having persistence of migraine [56].

Despite epidemiologic evidence in both adults and children supports the link between obesity and migraine, causal mechanisms remain poorly understood and speculative. Most likely the linkage between obesity and migraine is multifactorial and a variety of physiological, psychological, and behavioral mechanisms have been proposed. The inappropriate release of hypothalamic neuropeptides involved in energy balance regulation, as well as, the systemic pro-inflammatory state produced by the adipose tissue, might be advocated as possible causal factors for migraine development in obese individuals.

Different neuropeptides in hypothalamic control of feeding behavior have been proposed as key players in migraine pathophysiology. Serotonin through a diverse array of serotonin receptors impacts feeding behavior and satiety signaling. Except for transient rises in serotonin levels during headache attacks, low serotoninergic activity has been observed in migraine patients. This chronic decrease in serotonin release could contribute to increase appetite and weight gain [57–59]. Orexines are hypothalamic peptides that regulate arousal, wakefulness, appetite [60], neuropathic pain, migraine and cluster headache [61]. Since, low plasma levels of orexin-A have been detected in obese patients, it has been hypothesized that this orexin-A deficiency, by promoting the inflammation in the trigeminal system, might play a role in migraine genesis. A further neuropeptide is the calcitonin gene related peptide (CGRP), which is a proinflammatory substance playing an important role in both induction and perpetuation of the migraine attack [62, 63]. It has been observed that obese individuals show elevated levels of CGRP and that fat intake leads to further increases [64]. Chronically elevated circulating CGRP levels might decrease the trigemino-vascular activation threshold and therefore increase or trigger migraine attacks [65].

It is now established that adipose tissue is not only the primary site of storage of excess energy, but it also serves as an endocrine organ capable of synthesizing a number of bioactive substances, the adipokines, that regulate metabolic homeostasis [66, 67]. Imbalance between pro-inflammatory and anti-inflammatory molecules secreted by adipose tissue may play a critical role in the development of several obesity-related conditions [68].

Subclinical hypothyroidism (SH), characterized by serum TSH levels above the upper limit of the reference range, in presence of normal FT4 concentrations has distinctive features in childhood [69]. At variance from adults, in pediatric age SH seems to be less common (prevalence 1,7%) and is a benign and remitting condition, with a negligible risk of progression to hypothyroidism. Wasniewska et al. [70] found that 41.3% of children with idiopathic and mild SH between 5 and 10 mIU/l [4, normalized their TSH over time, while 58.7% remained SH and 12% increased TSH to > 10 mIU/l. None of these children showed any symptoms of hypothyroidism during 2-year follow-up [71]. Moreover, no alterations in cognitive functions, growth, bone maturation and body mass index status were recorded in children with idiopathic SH [72, 73].

However, in children with other disorders, including celiac disease, goiter and elevated anti-thyroglobulin autoantibodies, there is a deterioration of thyroid status over time, while in those with no underlying or associated disorders, there is a spontaneous normalization of thyroid function. High baseline TSH levels are probably the most powerful marker of persistent SH over time [74]. Turner (TS) or Down’s syndrome (DS) are chromosomopathies that are known to be linked with an increased risk of autoimmune thyroid diseases (AITDs) [71]. TS girls with hypothyroidism show a significant worsening of thyroid function over time and this trend is irrespective of both karyotype and other factors. DS children with SH might be prone to exhibit over time a phenotypic metamorphosis from hypothyroidism to Graves’ disease and to subsequently swing from hypothyroidism to hyperthyroidism [71].

A biochemical pattern of isolated hyperthyrotropinemia may be observed in around 7–10% of obese children. Although the pathophysiological mechanism responsible for SH in this population is not clear, it is postulated that the elevated levels of leptin in obese children can affect the hypothalamic regulation of TSH production. However, it was found that thyroid function may often normalize after weight loss. In obese children with longstanding SH, cardiovascular abnormalities and proatherogenic metabolic alterations may be sporadically seen. The major determinants of these alterations seem to be the duration of SH, that may be able to play a direct role on early atherosclerotic changes, not mediated by either visceral adiposity or other confounding factors [75]. In children with idiopathic SH, thyroid function monitoring should not be performed every 12 months. In HT-related SH children, thyroid function monitoring should be stricter: every 6 months. Because the association with either TS or Ds furtherly impairs the outcome of HT-related SH, thyroid function monitoring has to be stricter since progression from SH to overt hypothyroidism is more likely in these chromosomopathies.

In conclusion, SH is often self-limiting. L-T4 supplementation is not generally recommended in pediatric age, but it should be taken into consideration only in the cases with underlying HT or hypothyroid clinical symptoms, or thyromegaly, or very elevated TSH serum at entry. Recent studies comparing the effects of treatment versus no therapy in children with idiopathic SH seem to suggest that L-T4 therapy is unable to modify post-treatment outcomes of hyperthyrotropinemia and to prevent the risk of a further TSH increase after withdrawal [76].

### Gastroenterology

#### 1-acute appendicitis; 2-oral mucosal lesions; 3-constipation

The management of acute appendicitis at pre-school age has been reviewed by Almaramhy et al. [77] who emphasized that deferral in diagnosis is frequent because of vague presenting symptoms and poor communication skills of children. Several different conditions may be mimicked including acute gastroenteritis, mesenteric adenitis, intussusception, cholecystitis, constipation, respiratory tract and urinary tract infections, nephrolithiasis, hip arthritis, sepsis, meningoencephalitis. Therefore, young children are more disposed to develop complications. So, aiming at the decrease of morbidity and mortality rates linked to complicated appendicitis, it is required an early diagnosis. Computed Tomography scan is more useful than X-ray and ultrasonography. Several scoring systems may be helpful to reduce exposure to ionizing radiations. The standard of care for many years has been surgical appendectomy, but it carries with it several risks, including bleeding, wound complications, injury to surrounding structures, and the latent demand for reoperation. According to a recent systematic review, initial non-operative treatment of acute uncomplicated appendicitis in children was accompanied by similar rate of complications to children treated with appendectomy [78]. This procedure seems to be able to avoid an appendectomy in 62–81% of the children after one-year follow-up, although with low evidence. Similarly, a meta-analysis showed that antibiotics are effective in children with acute uncomplicated appendicitis without increasing the risk for complications [79]. The failure rate of antibiotic treatment is mainly caused by the presence of appendicolith. So, non-operative management of appendicitis in children can represent a well tolerated and reasonable treatment choice in selected patients [80] after discussion with caregivers on risks and benefits of both surgery and non-operative management.

Little is known about oral mucosa lesions (OML) which affects 30% of adolescents [81]. The most common lesions are aphthous ulcers, traumatic ulcerations, herpes simplex virus, geographic tongue, candidiasis and morsicatio buccarum. Some types of OMLs were shown in adolescents with systemic disorders (e.g. aphthous ulcers and celiac disease), as a consequence of the disease or as an outcome of the pharmacological treatment. In particular, multiple oral lesions may be an early expression of Crohn’s disease in children, preceding also the gastrointestinal manifestations [82]. Usually, orofacial features associated with Crohn’s disease are oral ulceration, lip, facial and buccal swelling, mucosal cobblestoning, angular and granulomatous cheilitis and gingivitis. Typically, oral lesions may be directly correlated with the involvement of the mucosa, appearing as granulomas, or may manifest specifically with stomatitis or aphthous-like ulcers, angular cheilitis, lip fissuring and gingivitis. Moreover, it must be considered that the occurrence of oral lesions correlates with the exacerbations. The paediatric dentists and gastroenterologists should be involved in the evaluation and follow-up of children suffering from Crohn’s disease.

Likewise, OMLs and gingival bleeding may reveal the evolution of liver failure in paediatric chronic liver diseases [83]. OMLs, such as candidiasis, telangiectasia, bald tongue, cracked strawberry lip, yellowish-brown gum discoloration, petechiae and gingival bleeding were related with the severity of liver dysfunction, coagulopathy, protein, bilirubin and creatinine levels and portal hypertension.

Furthermore, OMLs in children and adolescents may be the expression of malignancy, including mucoepidermoid carcinomas (22.4%), osteosarcomas (13.8%), squamous cell carcinomas (12.1%), and Burkitt’s lymphomas (12.1%) [84]. The most commonly affected sites were the palate (19%), mandible (13.8%), and maxilla (13.8%). Roughly half the patients were asymptomatic. Due to the rarity of these malignancies, it is fundamental to develop a cooperative networking among paediatricians, dentists, oncologists and surgeons about malignant oral tumours in children.

Functional constipation is a common gastrointestinal problem in childhood, rarely due to organic causes, including structural, endocrine, metabolic or allergic diseases [85, 86]. The pathogenesis of functional gastrointestinal disorders is unclear and probably multifactorial. The laxative treatment of paediatric functional constipation requires polyethylene glycol (PEG) as first choice, although it may change the intestinal microflora accelerating the peristalsis. Therefore, it has recently raised the interest on the efficacy of probiotics as supportive treatment for functional constipation. A probiotic mixture (PM), including *Bifidobacteria breve, infantis* and *longum* has been compared to the therapy with PEG alone in a paediatric population affected by FC [87]. In the short term, therapy of children with chronic functional constipation, PEG and PEG plus PM were similarly successful and safe. A long-term positive effect on constipation of the PM may justify further studies. Similarly, a systematic review found limited evidence on the effectiveness of probiotics in the management of constipation in children [88]. Although specific probiotic strains displayed effects on defecation frequency, none were useful on faecal incontinence or abdominal pain. In a randomized trial *Lactobacillus casei rhamnosus* Lcr35 was not more effective than placebo in the management of the FC among children < 5 years [89].

### Genetics

#### 1-fragile X syndrome

Fragile X Syndrome (FXS) is the most common known cause of inherited intellectual disability, diagnosed by the detection of an alteration of the Fragile X Mental Retardation-1 gene (FMR1), which maps at the Xq27.3 band, mainly by Triplet primed PCR [90]. FXS presents typical neurologic/neuropsychiatric features and involvement of multi-systemic characters, such as connective tissue signs, cardiac defects, gastrointestinal symptoms, pubertal macroorchidism, ocular anomalies, and obesity. Because of the physical, emotional and behavioural implications of the disease, patient management requires therapeutic strategies and social issues. FXS is associated with autism spectrum disorder in 50% of males and 20% of females [91]. This group of patients has a higher prevalence of seizures, sleep problems in childhood, increased behaviour complications, and higher use of α-agonists and antipsychotics. So, there is an urgent need of early identification of FXS individuals, to start an appropriate and timely interventions by medical and social services. A potential strategy to facilitate this procedure is the large-scale newborn screening [91]. However, a major limit of the screening may be represented by the actual lack of an effective targeted treatment when implemented early in infancy. It is also important to underline that several mothers, and other family members of children with FXS may be carriers of a premutation, less severe in clinical presentation but associated with different medical, cognitive/developmental, and psychiatric features [92]. So, an accurate diagnosis and a better understanding of the natural history of FXS is needed for better designing treatments, services and care. Pharmacological treatment is basically symptom-based to ameliorate behavior problems. Antipsychotics and selective serotonin reuptake inhibitors and lithium are commonly used. Non-pharmacological therapy including speech-language therapy, behavioral and physical therapy should be combined. A targeted therapy is now being studied that may radically change the destiny of the disease.

### Infectious diseases

#### 1- acute cerebellitis; 2- cytomegalovirus

An eleven-year retrospective multicentric study analysed clinical, neuroimaging and electrophysiologic features of acute cerebellitis (AC) and acute cerebellar ataxia (ACA) in children [93]. A total of 124 children were included, 118 with a diagnosis of ACA and 6 of AC. The most common signs and symptoms were broad-based gait disturbance, balance disorders, slurred speech, vomiting, headache and fever. Neurological sequelae were reported in 6 cases (5%), frequently associated to pathological magnetic resonance imaging or computed tomography. These long-term outcomes usually range from ataxia to mild tremor and the affected areas of cognition include spatial visualization ability, language skills, and concentration [94]. Although most cases are self-limited, these characters suggest performing an instrumental evaluation in all patients with AC/ACA at admission with the aim of preventing lethal complications and identifying patients at higher risk of neurological outcome [95]. A more aggressive therapeutic strategy and a closer follow-up should be achieved in this group of patients.

Cytomegalovirus (CMV) is the most frequent congenital infection, with a higher incidence in developing countries. CMV infection, although often asymptomatic at birth, may cause sensorineural hearing loss and neurodevelopmental disabilities in childhood [96]. Infants born to HIV-infected mothers are at higher risk of acquiring CMV infection [97]. CMV was positive in 23.2% among infants with in utero and 9.1% infants with intrapartum HIV infection recruited in a multi-centre clinical trial. Moreover, CMV was four-fold more prevalent among HIV-infected infants compared with uninfected infants born to HIV-infected mothers not receiving antiretrovirals during pregnancy. On the contrary, a cross-sectional Mozambican survey showed no differences in the prevalence of CMV infection among HIV-exposed and unexposed neonates [98]. Unfortunately, there is still a lack of preventive approaches and behavioural procedures for CMV infection during pregnancy. It is possible to treat infected infants with a well-tolerated antiviral drug, able to improve symptoms in childhood but not to eradicate the long-term sequelae.

### Neonatology

#### 1- drug use; 2- neonatal infections; 3-analgesia; 4- care givers; 5 -vitamin D

In developing countries, neonatal mortality is still high [99] compared to Western countries, particularly because of infections [100]. Thus, antibiotics are the most frequently prescribed medicines, followed by drugs for respiratory and nervous diseases in neonates, especially if with a gestational age below 32 weeks and/or birth weight < 2500 g. Almost 90% of the drugs used in neonatal intensive care units (NICUs) are off-label or unlicensed [101] and they are administered intravenously [102]. Girardi et al. have recently evaluated the drug use in neonates with low and extremely low birth weight at an Italian NICU [103]. They found that, compared to neonates with low birth weight, babies with very low birth weight were exposed to a higher number of drugs, mainly antibiotics, antifungals, and diuretics, and received more frequently associations of potentially nephrotoxic drugs. Moreover, most of them received more than 10 drugs during their stay at NICU. For most of these drugs, the risk-benefit profile is still not fully assessed in the neonatal population, and scanty evidence is available for their use in combination. Indeed, at present there is no standard profile of drug use for neonates in critical care, other than some efforts towards standardization, such as establishing local or general guidelines and publications like handbooks and formularies that are distributed worldwide. Further studies should explore the safety of the most used drugs in NICU patients, alone or in combinations, with particular focus on renal toxicity.

In hospitalized newborns, infections due to *Staphylococcus aureus* [104] and *Candida* species have become an increasingly important problem. In neonatal intensive care units (NICUs), *Candida* species are the third most common pathogen responsible of late-onset infections and entail high morbidity and mortality rates. Indeed, if babies survive to the infection, long-term neurological sequelae such cerebral palsy, blindness, hearing impairment, cognitive deficits, and periventricular leukomalacia may develop [105]. Garzillo et al. have recently investigated the phenotypic and genotypic features of *Candida parapsilosis* microbial isolates and evaluated the underlying clinical conditions associated with acquisition of the infection in 17 neonates at an Italian NICU [106]. During the 3 years of the study, *Candida parapsilosis* was responsible for 6 umbilical catheter- and 11 central catheter-associated bloodstream infections. Two isolates were resistant to fluconazole and intermediately susceptible to itraconazole. Birth weight, gestational age, and length of exposure to assisted ventilation represented independent risk factors for *Candida parapsilosis* infection both at the univariate and multivariate analysis. The colonization of neonatal skin and gastrointestinal tract is an important first step in the pathogenesis of the invasive disease [107]. Future studies aimed at better understanding the complex interactions between host risk factors and fungal virulence traits will hopefully allow to reduce the risk of systemic spread in neonates.

Exposure of newborns to uncontrolled and repetitive pain is common in neonatal intensive care units (NICUs), and may affect the infants’ pain perception, cognition, motor function, and brain development later in infancy. Therefore, the use of analgesia in neonates has increased largely in the last 25 years in order to reduce infants’ stress hormone response, time to recovery, and mortality rates [108]. In order to understand how much these practices have been implemented, the EUROPAIN survey has prospectively recorded round-the-clock bedside analgesia practices for all NICU admissions over 9 months. Lago et al. have recently analyzed the Italian data obtained for the EUROPAIN survey to document and compare current analgesia practices at 30 Italian NICUs versus other European centers and the current national guidelines [109]. Data from a total of 422 newborns (131 on invasive ventilation, 150 on non-invasive ventilation, and 141 on spontaneous ventilation) were collected. Analgesia practices were documented in the 35.3% of the total and varied considerably between the Italian NICUs as well as in other European countries. Strong analgesics, sedatives and mild analgesics were used in 32.5, 10.2, and 3.8% of the neonates, respectively. Illness severity, type of ventilation, and number of NICU beds were factors independently associated with analgesia use. Only one in four neonates was treated with boluses alone. The use of analgesia in the neonatal period is still poorly documented and few studies on this topic have been published up to now [110]. Further prospective longitudinal studies are warranted in order to check the performance of analgesia practices and their adherence to national guidelines.

The NICU environment is dramatically different from the maternal womb, as the neonate is exposed to variations in temperature, manipulation, different sensorial stimuli, noise, light, oxygen, and nutrients. These factors are a significant source of stress for the newborns and may negatively influence their neurodevelopmental outcomes [111]. At present, parents play only a supportive role in the NICU and thus keep feeling anxious and unprepared to care for their infant after discharge. On the other hand, family-centered care (FCC) has been increasingly emphasized as an important and necessary element in NICUs, as it expands parental presence in the NICU and requires that they have an active role in their newborn’s care [112]. De Bernardo and coworkers compared the satisfaction and stress levels of 48 parents in an FCC group versus 48 in a non-FCC program at an Italian NICU over one year [113]. They found not only that parents in the FCC group were more satisfied and less stressed than those in the other group, but also that infants in the FCC group showed a greater increase in body weight after 60 days. These findings prompt to support the implementation of FCC in NICUs, and also demonstrate that parents need to be involved in their child’s care. As parents play a key role in reducing their neonate’s stress, caregivers should encourage a greater parental involvement. Vitamin D insufficiency is common among children, particularly if born preterm [114] and/or affected by chronic diseases [115]. Preterm newborns are at particular risk of vitamin D deficiency as they are dependent on maternal vitamin D status during pregnancy and on nutritional intake after birth [116]. At present, there is still controversy about the adequate daily dose, duration and type of vitamin D supplementation that should be administered to preterm neonates. Cho and colleagues have recently evaluated the efficacy and safety of early supplementation with 800 IU of vitamin D in 49 very low birth weight infants [117]. They found that 88% of infants with baseline cord-blood levels of 25 (OH) D ≥ 10 ng/mL achieved vitamin D sufficiency at 36 weeks of postmenstrual age, but the proportion decreased to 65% in those with baseline cord-blood levels of 25 (OH) D < 10 ng/mL. Only three infants showed laboratory signs of hypervitaminosis D. These findings confirm and expand those from previous studies [114] as they demonstrate that the response to vitamin D supplementation depends on the baseline vitamin D stores at birth. As Vitamin D is essential for the normal development of skeletal and extra-skeletal systems, low 25 (OH) D levels are an issue for preterm VLBW infants, calling for early nutritional interventions. Further research is needed to figure out the exact dose to safely meet target levels without overcorrection.

### Nephrology

#### 1- solitary kidney; 2- nephrotic syndrome

During the last decades the potential role of solitary kidney (SK) in promoting systemic hypertension, proteinuria and glomerulosclerosis has been widely studied. Lubrano and colleagues [118] assessed mid- and long-term outcome of children with congenital SK (CSK) or acquired SK (ASK). Patients with ASK were significantly older than those in the CSK group [119]. Glomerular filtration rate, proteinuria, and blood pressure of each patient were collected from medical records at the time of first admission and at 14 years of age and there was no difference between groups. Blood pressure values fell into the normal range for most patients without any difference between the two groups. At the end of the study period, the percentage of patient with blood pressure > 90th centile had significantly increased, A significant increase of prehypertension in the CSK group and a significant increase of hypertension in the ASK group were found. In the light of these results, children with SK are potentially predisposed to a higher risk of hypertension and prehypertension in later life. Careful monitoring throughout childhood is therefore mandatory to prevent progression to more serious complications.

Regarding nephrotic syndrome (NS) in children, this year has been issued the Consensus on clinical management by the Italian Society for Paediatric Nephrology [120]. For the first episode of NS authors recommend a daily glucocorticoid therapy with prednisone (PDN) or its active metabolite, prednisolone at 60 mg/m^2^/day (maximum: 60 mg/day) either in a single daily dose in the morning or in two divided doses as the efficacy is comparable. A 12-week treatment regimen including 6 weeks of prednisone at 60 mg/m2 per day plus 6 weeks at 40 mg/m^2^ (maximum: 40 mg/day) in a single dose on alternate days was recommended. The first relapse requires PDN, 60 mg/m2/day until urine protein levels normalize for 5 days. The dose is then reduced to 40 mg/m2 alternate days for 4 weeks. Sodium restriction to a level < 1–2 g or < 35 mg/kg daily and fluid restriction is recommended in case of mild oedema. Moderate oedema may require loop diuretics in addition to salt and water restriction. In case of severe/refractory oedema unresponsive to oral or i.v. loop diuretics a thiazide diuretic and albumin infusions should be administered. The complications of childhood NS are associated with disease activity and therapy. Infections are a common complication but there are no data supporting the efficacy of prophylactic antibiotic therapy. Thromboembolism develops in 2 to 5% children with NS. A thrombophilia screening is recommended only in patients with risk factors for thromboembolism such as known abnormalities of pro-thrombotic coagulation factors or a family history of thrombotic events at a young age (< 50 years). Prophylactic anticoagulation/antiplatelet should be considered in the setting of concomitant cardiovascular abnormalities, thromboembolism history, an underlying hypercoagulable condition beyond NS, steroid-resistant NS, and the presence of a central venous catheter. Prophylactic proton pump inhibitors (PPIs) during steroid therapy is necessary only in patients with gastric symptoms or with other risk factors for gastrotoxicity. At the time of first presentation, 15–20% of children with NS fail to achieve complete remission within 4 weeks of corticosteroid therapy and are classified as having steroid-resistant nephrotic syndrome (SRNS). These children are at increased risk for progression to end-stage kidney disease. Because of this risk and the potential utility of histology for therapeutic decision-making, kidney biopsy should be performed. The most common finding on kidney biopsy in patients with SRNS is a focal segmental glomerulosclerosis (FSGS). Although a definitive pathogenic mechanism has not been identified it seems that some forms of FSGS may be due to genetic mutations of podocyte proteins [121]. Five particular gene mutations are involved in nonsyndromic FSGS, each of these genes play a role in the development, migration, basement membrane interaction, and regeneration of the podocyte. It is also possible that a circulating serum factor may cause SRNS. Accordingly, proteinuria develops immediately after renal transplantation in patients with FSGS. Dogra and colleagues [121] suggested the need for genetic testing in a patient with NS, aged less than 2 years with positive family history of nephrotic syndrome, consanguinity, a steroid-resistant course, and histopathologic findings of FSGS or diffuse mesangial sclerosis on renal biopsy.

Therapy for SRNS includes immunosuppressive, immunostimulatory and non-immunosuppressive drugs. The more commonly used immunosuppressive drugs are calcineurin inhibitors, mycophenolate mofetil (MMF), pulse intravenous methylprednisolone, and cytotoxic agents. In a randomized controlled trial, Sinha et al. [122] showed that in children with idiopathic SRNS 61% of patients achieved complete remission and 38% partial remission after treatment with tacrolimus for 6 months. Then, children were randomized to receive tacrolimus or MMF both associated to prednisolone on alternate days for 12 months. At the end of 12 months follow-up, rate of patients with a complete and partial remission was significantly higher in tacrolimus group (90.3%) compared with MMF group (44.8%). Children treated with MMF had a significant higher incidence of relapses and they needed a significant higher dose of prednisolone. Rituximab, a monoclonal antibody directed against the cell surface antigen CD20 expressed on B lymphocytes, has been used in children with idiopathic NS resistant to steroids and to all immunosuppressive agents (approximately 1–3% of cases). However, there are promising data but no clear evidence that rituximab is effective for patients with refractory SRNS [123]. In patients with complicated frequently relapsing/steroid-dependent NS, rituximab does not cure the renal disease, as most patients relapsed during follow-up and need to continue immunosuppressive agents (such as CsA or MMF) after rituximab treatment to get long-term remission [124]. Moreover, rituximab increases the risk of infection, mostly viral, because of B-cell depletion [125]. Fujinaga and colleagues [126] reported complete remission after repeated administration of Rituximab in a child with refractory SRNS due to FSGS. Two months after the last administration of Rituximab the patient developed severe hypogammaglobulinemia requiring i.v. gammaglobulin. Novel agents are under investigation, but their safety and efficacy have not yet been determined.

### Neurology

#### 1-sleep habits; 2-Ataxia; 3-child abuse and neglect; 4- Fabry disease; 5- congenital myopathies

Several researches have demonstrated that non-sufficient sleep in childhood is associated with immediate and long-term adverse consequences for mental and physical health. A questionnaire study investigated sleep habits of a large cohort of Italian children between 1 and 14 years to assess their relationship with evening activities performed before bed time [127]. Younger children slept longer than the older ones. The mean sleep duration was 11.5 h in 1 to 3 years old children vs 9.0 h in 10 to 14 years old but only 66.9% of them got sufficient sleep as recommended by National Sleep Foundation [128]. The use of display devices before sleeping was a significant predictors of shorter sleep duration. Similarly, having TV in the bedroom, being only child, bottle use habit and a history of sleep disorders during the first year of life negatively influenced optimal sleep conditions. These data agree with the literature data worldwide of the last decade. Tamura and colleagues [129] found a higher incidence of insomnia and depression and a shorter sleep duration in adolescent using mobile phone for 5 h or more per day. Females had a longer use of mobile phone than males. In particular, hours spent using a mobile phone for social networking sites and online chat were linked with depression, while internet searching and playing video games were not.

In 2334 Canadian children [130] a longer sleep duration was associated with frequent reading a book in the bedroom and absence of electronic devices, on the contrary use an electronic device before to sleep was associated with shorter sleep duration and shorter total time in bed. Children with a frequent use of computer and TV during the hour before sleep had 20% odds of being overweight and double odds of being obese. Results were similar for cell phones use. Surprisingly, children having a TV or a video game in their bedroom were at risk of overweight and obesity even if they did not use them before sleeping. A study conducted in USA among low-income preschoolers found an inverse relationship between sleep duration and body mass index (BMI) [131]. Future studies investigating the association between sleep duration, electronic devices use, and BMI are needed to clarify this relationship and the risk of overweight and obesity particularly in high-risk population.

Ataxia is a manifestation of a multitude of disease processes, and an underlying etiology needs to be investigated. Detecting ataxia can be challenging specially in early childhood but simple signs and maneuvers can help to recognize ataxia. In a recent review, Pavone and colleagues [132] provided a general approach to assessing and managing the patient with ataxia, they also reported the background and common etiologies of ataxia. Unsteadiness of gait with loss of balance is the more common symptom in younger children. Older children often feel insecure and have to hold onto the wall and walk with feet apart. In children over 3 years typical maneuvers such as finger to finger or finger to nose, and rapid alternating hand movements can be performed to explore coordination, while dysmetria can be tested through heel-knee test. Children with ataxia tend to fall down with closed eyes (Romberg sign positive) and cannot hold a glass of water without letting the water fall from the glass. They suggest different ways to classify ataxias but conclude that, in children, the most useful considers time trend. Ataxia may be distinguished as being acute, intermittent and recurrent, chronic-non-progressive and chronic-progressive. The most common etiologies of acute ataxia in children are excessive drug ingestion, drug intoxications and post-infectious cerebellitis, while basilar migraine is the most common cause of intermittent ataxia. But ataxia can also be a sign of rare and more complex diseases as hereditary ataxias. The most well-known of the inherited ataxias includes ataxia-telangiectasia (AT), a rare, neurodegenerative, multiorgan, autosomal recessive disorder causing severe disability. Because of his rarity, the diagnosis of AT is often delayed but it can be made earlier with serum alpha feto-protein (AFP) measurement, a readily available and inexpensive test for all toddlers and children with undiagnosed chronic or progressive ataxia [133]. Children with AT suffer from increased mortality because of lymphoreticular malignancy, infections of the respiratory system, and various complications. The gene mutated in AT, ATM (ataxia telangiectasia mutated) gene, encodes a large protein kinase. The nature of the immune deficiency is highly variable due to a plethora of mutations of the ATM gene. Some children have a variant AT in which a residual kinase activity leading a milder clinical phenotype, with different immunoglobulin patterns and a longer survival compared to classical AT patients. Life expectancy seems to correlate to some immunoglobulin pattern alterations (IgG2 deficiency or hyper IgM phenotype with hypogammaglobulinemia) [134].

In recent decades, the concept of child abuse and neglect has evolved [135]. Children of the new era are increasingly stressed because of changes in living conditions, modern family types, loss of a parent or increasing expectations from parents, teachers or other family members. Increasingly large numbers of children and adolescents have been forced to migrate across the world for several reasons. Mental health of these children is of particular concern because of their experiences. The exposure to organized violence and threats arising from religious, cultural, and political differences, or territorial disputes make them vulnerable to several and cumulative risks to their physical, emotional, and social development. Another class at risk are children in long-term foster care. Several evidences show that children in care have significant developmental, behavioral, and emotional problems. Parental divorce/separation is among the most commonly endorsed adverse childhood events and is another highly prevalent risk factor associated with high rates of mental health problems for youth and increased later risk of alcohol dependence across adolescence and early adulthood [136].

Soares and colleagues [137] conducted a comparative study to examine the association between parental divorce during childhood and cardiometabolic risk factors in youth, using data from 2 cohort studies in contrasting contexts (the United Kingdom and Brazil). In both cohorts, cigarette daily smoking was more frequent among adolescents whose parents separated before age 18 years. There was no interaction between age at parental separation and parental relationship conflict and cardiometabolic risk factors. A higher risk of harmful alcohol use was seen in Brazilian cohort but not in English one. Several studies of child maltreatment and sexual risk behavior have shown that males and females who experienced any kind of sexual abuse had significantly increased odds of engagement in risky sexual behaviors, but a recent study revealed that other forms of maltreatment can increase risky behaviors. Thompson and colleagues [138] showed that maltreated youths continue to be at high risk for engaging in behaviors that may start a trajectory of problematic sexual behaviors. Clinical and prevention programs should target adolescents with a history of maltreatment and pediatric clinicians have a responsibility to screen at-risk youth for maltreatment experiences. A recent review [139] focused on possible means to screen for child physical, sexual, and psychological abuse and neglect. One screening tools for physical abuse, called the “Escape Form” was applicable to children of any age. Limit of this tool is the use only in emergency situations. Only one screening instrument was found for sexual abuse but it was not approved by the International Association of Forensic Nurses and no screening tool was found to identify psychological abuse and neglect in children. These findings show that further efforts are needed to develop an effective instrument that can identify and prevent child maltreatment and improving child welfare services.

Fabry Disease (FD) is an X-linked multisystemic, lysosomal storage disorder caused by deficiency of α-galactosidase A activity, with consequent accumulation of globotriaosylceramide (Gb3) and related glycosphingolipids, such as globotriaosylsphingosine (lyso-Gb3) in lysosomes. The main complications of FD are more prominent after the age of 30 when kidney, heart and/or cerebrovascular disorders appear. Regarding cardiac involvement, deposits of neutral glycosphingolipids within cardiocytes lead to myocyte hypertrophy and fibrosis causing conduction abnormalities, left ventricular hypertrophy (LVH) or valvular fibrosis. Patients with manifestations limited to the heart have been reported as a disease variation. Csányi et al. [140] have found a novel Ile239Met mutation in α-galactosidase A gene in a family with a predominant cardiac phenotype of Fabry disease. In this family, 6 individuals carried the Ile239Met mutation of whom 3 members manifested the cardiac phenotype of hypertrophic cardiomyopathy and 2 other family members showed LVH. Interestingly, 4 of the 5 with cardiac involvement were female individuals. This finding is unusual for cardiac FD since the disease shows X linked inheritance.

Wilson et al. [141] analyzed electrocardiographic and clinical findings in pediatric FD patients. The most common arrhythmia was sinus bradycardia (23%), followed by ectopic atrial rhythm (12%) and premature atrial contraction (8%). No episodes of nonsustained ventricular tachycardia, supra ventricular or ventricular tachycardia, atrial fibrillation and no PR or QTc intervals abnormalities were found. Only 1 female developed a first-degree atrioventricular block during follow-up. Chest pain (35%) and palpitations (23%) were the most common symptoms. Echocardiography detected aortic root dilation in 3 patients, one of them also had concurrent mild aortic insufficiency. These findings show that cardiological involvement in Fabry disease at the beginning is limited and this results in clinical symptoms, abnormalities of conduction and structure largely heterogeneous. In cardiac involvement in a Fabry’s patient, enzyme replacement therapy (ERT) should be started before myocardial fibrosis has developed to achieve long term improvement. Many efforts are needed in order to identify early cardiac alterations to increases the chances of patients to receive a proper therapy and suggest the best age for ERT onset. A recent study suggests that blood LysoGb3 concentrations measurement could aid to define a threshold concentration for new preventive and therapeutic approaches [142].

After the diagnosis of FD in a 11 years old child, the authors detected the same disease in his little brother, 14 years younger, during neonatal period. The younger brother underwent LysoGb3 analysis at 2 days of life. Blood LysoGb3 concentration obtained from the asymptomatic second-born were compared with serum levels from the symptomatic first-born: there was a 5-fold increase from the neonatal period to childhood; moreover, at five months of age the concentration of LysoGb3 reached 40% of the value observed in the symptomatic phase moreover. They also noted that during neonatal period the value was 15 times higher than children without FD. These findings have demonstrated the key role of LysoGb3 as potential mean to diagnose FD. The authors documented an early plateau during infancy which precedes the symptomatic phase. This can help to identifying a metabolic threshold may lead to new preventive and therapeutic approaches.

First specific therapy for Fabry’s disease is enzyme replacement with recombinant human galactosidase A that provides an exogenous source of deficient enzyme in patients with this progressive disorder. Two drugs are now commercially available: agalsidase alpha and agalsidase beta. A global shortage of agalsidase-β between 2009 and 2012 caused many patients worldwide to be switched from agalsidase-β to agalsidase-α. Skrunes and colleagues [143] described the effects of switch from agalsidase-β 1.0 mg/kg/every other week (eow) to agalsidase-α 0.2 mg/kg/eow in serial kidney biopsies in 3 patients. Each patient was reassessed by renal biopsy after 5 years of agalsidase-β and 3 years of agalsidase-α. Biopsies after 5 years of agalsidase-β 1.0 mg/kg/eow showed marked clearing of globotriaosylceramide (GL3) from mesangial and endothelial cells and partly cleared podocytes in all patients. After 3 years of agalsidase-α on a lower dose (0.2 mg/kg/eow) there was a reaccumulation of GL3 in podocytes, but not in the mesangium or endothelium. When agalsidase-β was again globally available, one of the patients was reswitched to agalsidase-β with a consequent decrease of podocyte GL3 deposits. These results point out the importance of dose in enzyme replacement therapy (ERT) in preventing irreversible kidney damage.

Congenital myopathies (CM) are a heterogeneous group of muscular disorders characterized by the presence of specific morphologic features on skeletal muscle biopsy. More than 25 genetic causes of CM and several types of mutations within the same gene have been found. Cassandrini et al. [144] focused on clinical and genetic forms of CM in order to facilitate histological and imaging diagnosis. Based on the results of muscle biopsy can be recognized five forms of CMs: nemaline myopathy (NM), core myopathy, centronuclear myopathy, congenital fiber-type disproportion myopathy, myosin storage myopathy. The morphological hallmark of NM is the presence of nemaline bodies or rods. It is usually considered the most usual form of congenital myopathy, although central core disease can have higher prevalence due to longer term survival. Rod bodies may be present in most muscle fibers, and may occupy over half of a fiber’s volume. In a rare subtype of NM nemaline bodies are present inside myonuclei. There are currently ten known genetic causes of NM, of which the nebulin (NEB) gene, inherited in an autosomal recessive manner, and actin (ACTA1) gene inherited in an autosomal dominant (90%) or recessive (10%) way, are the most important. Core myopathy is an autosomally inherited muscle disorder characterized by the presence of cores in muscle fibers, they can be single or multiple in the same muscle fiber. The disease is associated with defects in the RYR1 gene located on the chromosomal region 19q12–13.2. There are some families in which no abnormality has been found in the RYR1 gene, thus suggesting that mutations in other unidentified genes may be responsible in a minority of families. The other forms of CM are relatively rare. Identification of the true gene mutation is indispensable for a better clinical stratification. However, it is difficult establishing a correct genetic diagnosis for CM. New clinical and histological phenotypes are still being identified for some of the known disease genes and this for many reasons. Furthermore, mutations in different genes can cause many of the histological patterns, and mutations in a single gene can result in various histopathological abnormalities. New genetic approaches are being devised, such as full genome sequencing, which are likely to overcome this barrier and allow even the rarest genetic disorders to be genetically characterized.

As seen, the most important diagnostic clues of congenital myopathies are the presence of particular abnormalities of muscle fiber architecture on muscle biopsy. Witting et al. [145] reported a comprehensive analysis of the prevalence, genotype, and phenotype of CM in patients 5 years and older in Denmark. After identifying all registered Danish patients with a diagnosis of CM aged older than 5 years, comprehensive clinical, histopathologic, and genetic investigations were performed. Of 82 patients included in the study, 41 had specific histology. A core myopathy was detected in 14 (17%), a centronuclear myopathy (CNM) in 15 (18%), and nemaline myopathy (NM) in 12 (15%). The other patients had undistinctive features on biopsy. A genetic etiology was reached for 83% of patients with histopathologic features of core myopathy CNM, or NM but only for 29% of patients with unspecific histopathologic features (56% of overall cases). The authors also found that the prevalence of CM in Danish patients older than 5 years was 2:100,000 significantly lower than previous studies. This may be due to the exclusion of pediatric patients who have succumbed to their illness prior to age 5. This approach allowed for a unique prospective assessment of this patient group that has not been studied previously in CM.

Congenital NM due to mutations in troponin T1 (TNNT1) has hitherto only been described as a result of a single homozygous nonsense founder mutation in patients of Amish origin and in other individuals with 4 different recessive mutations. Konersman and colleagues [146] described a novel heterozygous missense mutation of TNNT1 responsible of NM. Blood samples from members of an extended family with Ashkenazi Jewish ancestry underwent Sanger sequencing for TNNT1. The history of the family revealed multiple cases of myopathy in three different generations and a mild phenotype with considerable clinical heterogeneity. Skeletal muscle biopsies showed severe type 1 fiber hypotrophy, with many red/purple-staining rod-like structures exclusively in type 1 fibers. Genetic testing showed the presence of a novel heterozygous missense variants in exon 9 of the gene TNNT1 c.311A > T. This mutation was transmitted in an autosomal dominant fashion whilst, all of the previously described mutations were autosomal recessive cases of TNNT1-related NEM. This report expands the phenotype associated with TNNT1 mutations and may lead clinicians to suspect NEM5 outside the Amish population particularly in mild cases of NM with nemaline rods and an autosomal dominant pattern of inheritance.

### Pulmonology

#### 1-oral corticosteroid; 2-prevention of bronchiolitis

Acute respiratory diseases are still a leading cause of childhood morbidity and mortality. Both inhaled and systemic corticosteroids have been widely used for the treatment of many acute respiratory illnesses associated with airway inflammation. Recently, Cutrera et al. [147] have reviewed the role of oral corticosteroids in the treatment and management of pediatric bronchiolitis, wheezing, asthma, and croup. Bronchiolitis is the most common cause of acute lower respiratory infection in the first year of life, with the highest rate of hospitalization occurring in infants aged less than 3 months, preterm babies, children with chronic lung, heart or neuromuscular diseases, and infants with immunodeficiency. Moreover, bronchiolitis due to respiratory syncytial virus, particularly if severe, increases the risk of recurrent wheezing and asthma later in life. Oxygen supplementation and hydration remain the mainstay of treatment, while there is no evidence supporting the usefulness of inhaled or oral corticosteroids in routine practice, as they do not decrease the incidence or duration of hospitalization, neither do they improve the short- and long-term prognosis. Preschool wheezing is a heterogeneous condition that usually resolves by school age but may also represent the first manifestation of asthma in some children. Therapeutic strategies for recurrent wheezing are tailored to the frequency and severity of clinical manifestations [148]. Evidences supporting early family/carer-initiated oral corticosteroids for home management of exacerbations are weak, and moreover this treatment is not indicated for preschool children with mild exacerbation of viral wheeze, while it should be reserved to patients with severe wheezing exacerbations. Asthma is one of the most common reasons for urgent care, emergency department visits and hospitalizations. The primary goal of asthma management is to achieve disease control, as asthma not responding to treatment may result in significant morbidity, including risk of future exacerbations and progressive loss of lung function [149]. Oral corticosteroids are commonly used in case of moderate and severe asthma attacks [150], since they result in fewer and shorter hospitalizations, more rapid improvement in lung function, lower rate of relapse after discharge from the emergency department, and reduced need for SABA. A liquid formulation should be preferred to tablet in children. Laryngotracheitis, or viral croup, is often mild and self-limiting, and usually resolves without any active intervention. Oral corticosteroids are the treatment of choice for children with mild-to-moderate croup, while inhaled corticosteroids and nebulized epinephrine are indicated for children with severe respiratory distress. One of the most frequent medical problems affecting newborns and infants is the occurrence of respiratory infections [151]. Worldwide, bronchiolitis is the main cause of lower respiratory infection in infants. Several studies have investigated the relationship between breastfeeding and bronchiolitis. The advantages of breastfeeding are largely documented across different health outcomes in both developed and low-income countries. In particular, the positive effects of breastfeeding seem to be clear in reducing the risk of infectious diseases in infants. During the transitional period from prenatal to postnatal existence, newborns are highly susceptible to infections because of their immunological immaturity. After the fetus has developed in the sterile protective environment within the uterine cavity, at birth the immune system undergoes a maturation process that is modulated by exposure to a variety of maternal and environmental infectious and non-infectious agents. Among them, many anti-microbial, anti-inflammatory and immunomodulatory agents present in human milk stimulate the maturation of the immune system conferring an overall protection to the newborn. It has been widely discussed that breastfeeding protects infants against respiratory infectious diseases, however the results of the studies conducted so far are controversial. Indeed, the composition of breast milk significantly affects children’s immune function and development by providing several soluble factors. Li et al. [152] assessed the concentrations of some immunomodulatory constituents of human milk from mothers of 20 infants with bronchiolitis and 11 healthy controls. They found that breast milk IgG levels were significantly lower in mothers of bronchiolitis patients than in those of controls, while there were no significant differences in total T, B, or NK cells between the two groups. Indeed, it has been previously published that infants receiving exclusively or non-exclusively maternal milk have an overall lower risk of bronchiolitis occurrence, with similar levels of protection for both groups [153]. Taken together, these results may be explained by the wide properties of maternal milk, that has both a direct and an indirect action against pathogenic agents. IgG contained in breast milk may be absorbed by the breastfed infant and modulate his/her immune responses, which could play a significant role in the resistance against bronchiolitis.

Bronchiolitis is one of the most relevant health burdens in infants and young children worldwide. Respiratory syncytial virus (RSV) is the most frequent pathogen involved, with about 34 million new cases in children younger than 5 years, 3.4 million admissions to hospitals, and about 199,000 deaths per year, predominantly in the developing world [154]. In 1998, the monoclonal antibody palivizumab was approved for the prevention of severe RSV infection in infants with bronchopulmonary dysplasia and in children born at ≤35 weeks of gestational age. Subsequently, Italian Guidelines recommended palivizumab prophylaxis also for infants born at 29–35 weeks of gestational age and a chronological age ≤ 6 months at the beginning of the epidemic season, in presence of risk conditions predisposing to severe infections and/or need for hospitalization, such as attendance of the child in a community setting and/or presence of one or more cohabitees younger than 5 years. However, in 2014 the American Academy of Pediatrics recommended against the use of RSV prophylaxis in all children born at 29–35 weeks unless the infant has an underlying health condition such as bronchopulmonary dysplasia or congenital heart disease. Following these changes, in September 2016 the Italian Drug Agency decided for the total financial coverage of the palivizumab prescription to healthy preterms only if they were < 29 weeks of gestational age at birth and if are aged ≤12 months at the beginning of the RSV epidemic season. Capizzi et al. evaluated the possible impact on the incidence and severity of RSV bronchiolitis after the new prescription limitation [155]. They found that, compared to the 2 years before the changes made by the Italian Drug Agency, during the year after the prescription limitation start there was a significantly higher proportion of admissions to hospital for RSV bronchiolitis in infants < 36 weeks of gestational age at birth and of patients needing high flow nasal cannula ventilation. These findings have been recently confirmed by an American study, showing that RSV hospitalization rates increased by 2.7-fold in infants aged 29–34 weeks of gestational age at birth [156]. Taken all together, these results are of clinical concern and point towards the need for a re-evaluation of the role of palivizumab prophylaxis in infants aged > 29 weeks of gestational age at birth.

## Conclusion

The year 2016 has been an exciting year in many areas of pediatrics. The Italian Journal of Pediatrics is not devoted to a single pediatric specialty and new data could also be found in other journals. However, the 2017 content has provided a great quantity of clinically useful information that improve our understanding in many fields. Basic studies have analyzed steps toward reducing neonatal mortality in developing countries and improving sleep habits. Indications on risk factors for *Candida parapsilosis*, migraine and hypothyroidism have been reported. Progresses in our understanding of bronchiolitis have highlighted the role of human breast milk and of palivizumab in prevention of RSV infection. Novel observation on assessment of SPTs for the identification of children with cow’s milk allergy, definition of obesity and differentiation of OML have been presented to facilitate diagnosis. Consensus on AIT and on management of nephrotic syndrome are an important support to improve quality of care. Recent advances have also included many observations that can contribute to a better approach to diagnosis and treatment of children with acute appendicitis, ANCA-associated vasculitis, ataxia, FXS, congenital CMV infection, CM, FD and hypertension in SK. Insights have emerged on etiopathogenic mechanisms and clinical aspects of less frequent illnesses such as acute cerebellitis and SSSS in newborns that improve the current management. Progresses have emphasized a need to better support caretakers for preventing abuse and backing parents of infants admitted at NICU. Important advances in treatments have included drug use among preterm neonates, sedation and analgesia in NICU, Vitamin D supplementation, TCS phobia and benefits from probiotics in allergic rhinitis and chronic functional constipation. We hope this review would be useful to give guidance to at once ameliorate patient care.

## Abbreviations

AC: Acute cerebellitis
ACA: Acute cerebellar ataxia
AFP: Alpha feto-protein
AIT: Allergen-specific immunotherapy
AKI: Acute kidney injury
ANCA: Anti-neutrophil cytoplasmic antibody
ASK: Acquired solitary kidney
AT: Ataxia-telangiectasia
ATM: Ataxia telangiectasia mutated
AVA: ANCA-associated vasculitis
BMI: Body Mass Index
CM: Congenital myopathies
CMV: Cytomegalovirus
CSK: Congenital solitary kidney
EGPA: Eosinophilic granulomatosis with polyangiitis
EOW: Every other week
ERT: Enzyme replacement therapy
FD: Fabry Disease
FRNS: Frequently relapsing nephrotic syndrome
FSGS: Focal segmental glomerulosclerosis
FXS: Fragile X syndrome
Gb3: Globotriaosylceramide
GPA: Granulomatosis with polyangiitis
LVH: Left ventricular hypertrophy
Lyso-Gb3: Globotriaosylsphingosine
MMF: Mycophenolate mofetil
MPA: Microscopic polyangitiis
NICU: Neonatal Intensive Care Unit
NM: Nemaline myopathy
NS: Nephrotic syndrome
OML: Oral mucosa lesions
PDN: Prednisone
PPIs: Proton pump inhibitors
RSV: Respiratory syncitial virus
SDNS: Steroid-dependent nephrotic syndrome
SK: Solitary kidney
SPT: Skin prick tests
SRNS: Steroid-resistant nephrotic syndrome
TCS: Topical corticosteroids
TNNT1: Troponin T1
WHO: World Health Organization
